# Hybrid 3D Printing of Interfacial Polyelectrolyte Complex Formed between Hyaluronic Acid and Poly-L-lysine Hydrogels

**DOI:** 10.1021/acsomega.6c06625

**Published:** 2026-08-27

**Authors:** Yi-Chen Huang, Ming-Chia Li

**Affiliations:** † Department of Biological Science and Technology, 34914 National Yang Ming Chiao Tung University, Hsinchu 300, Taiwan; ‡ Center for Intelligent Drug Systems and Smart Bio-Devices (IDS2B), National Yang Ming Chiao Tung University, Hsinchu 300, Taiwan

## Abstract

Hyaluronic acid (HA), a principal component of the extracellular matrix (ECM), exhibits excellent biocompatibility, high water-retention capacity, and inherent biodegradability. α-Poly-*L*-lysine (PLL), a cationic polypeptide with favorable biocompatibility, is widely employed for surface modification to promote cell adhesion and proliferation. Electrostatic interactions between oppositely charged polyelectrolytes enable the formation of interfacial polyelectrolyte complexes (IPC) in the absence of chemical cross-linking agents. In this study, IPC hydrogels were fabricated from HA and PLL and processed via a combination of hybrid and gravity-compensated embedded three-dimensional (3D) printing techniques to generate both two-dimensional (2D) and 3D scaffolds for tissue engineering applications. Using this approach, anisotropic 2D membranes with submicrometer groove topographies were produced, together with mechanically stable 3D fibrous architectures. The resulting IPC hydrogels retained structural integrity under aqueous conditions, demonstrating high morphological controllability and highlighting their substantial potential as scaffolding materials in tissue engineering.

## Graphical abstract

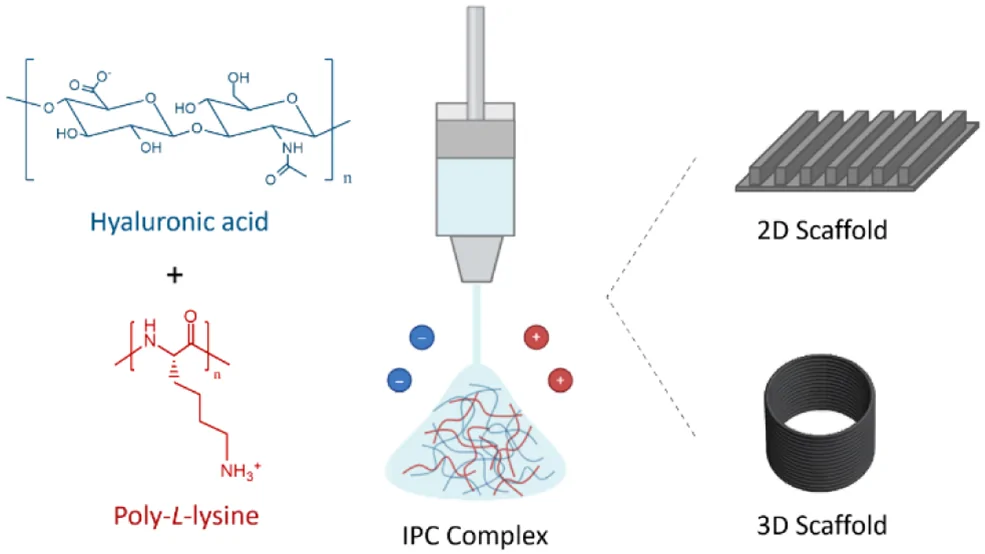

## Introduction

Various proteins and glycosaminoglycans (GAGs) interweave to constitute the structural architecture of the extracellular matrix (ECM).^1^ Cell surface receptors interact with the ECM to bridge the extracellular space and cytoskeleton, driving bidirectional signaling that allows cells to dynamically adapt and regulate tissue development.^2^ Furthermore, GAGs can bind proteins to form proteoglycans, including perlecan, syndecans, and glypicans.^2^ Among ECM components, collagen is the most abundant protein, inhabiting connective tissues and forming triple-helical scaffolds that maintain structure and regulate diverse cellular functions.^3^ GAGs are linear polysaccharides such as heparan sulfate and hyaluronic acid that play important roles in various biological processes.^4^

As a key constituent of the ECM, hyaluronic acid (HA) exhibits remarkable biocompatibility, superior water-holding capacity, and natural biodegradability.^5^ HA is widely distributed in connective, epithelial, and neural tissues.^6^ In the nervous system, HA is enriched in neural stem cell niches and perineuronal nets, where it regulates progenitor cell behavior, synaptic plasticity, and extracellular matrix architecture.^7^ In wound dressing applications, HA has also been shown to accelerate the healing of burns, epithelial surgical wounds, and chronic wounds.^6^ Structurally, HA is a high-molecular-weight polysaccharide consisting of repeating units of *D*-glucuronic acid and N-acetyl-*D*-glucosamine, interlinked via alternating β-1,4 and β-1,3 glycosidic linkages, exhibiting a molecular mass that can reach up to 10 MDa.^8^ In addition to these well-known functions, HA also alleviates gastro-esophageal reflux and promotes repair of the esophageal wall.^9^

Cationic *L*-lysine is one of the most abundant essential amino acids in the human body and plays a critical role in various physiological processes.^10^ Poly-*L*-lysine (PLL) is a polymeric material synthesized through the polymerization of l-lysine monomers.^11^ Owing to its low immunogenicity, antimicrobial activity, excellent biocompatibility, and biodegradability, PLL has attracted significant attention and has been widely utilized in biomedical and pharmaceutical applications.^12^ Due to the protonation of free amine groups on its side chains under physiological conditions, PLL exhibits a cationic nature.^13^ Consequently, aqueous solutions of PLL are typically alkaline, with a p*K*_a_ of approximately 9.0. Positively charged PLL demonstrates significant bioactivity and multifunctionality in various biomedical applications.^14^ PLL has therefore been developed as a functional biomaterial, with its activity largely attributed to its cationic properties.^15^ Through electrostatic interactions between positively charged PLL and negatively charged guest molecules, PLL has been extensively investigated as a candidate material for nanocarriers, surface coatings, and antibacterial applications, including biofilm dispersion and membrane disruption.^16^

Nanoimprinting technology has been widely utilized in tissue engineering and biomedical applications for the fabrication of micro- and nanoscale surface features. These nanoimprinted architectures, including nanofibers, nanowires, nanoholes, and nanopillars, are capable of directing cell growth and promoting cellular differentiation.^17^ Polyelectrolyte systems based on nanofibrous or hydrogels composed of hyaluronic acid (HA) and α-poly-*L*-lysine (PLL) can encapsulate therapeutic molecules through electrostatic interactions, serve as drug delivery carriers, and enable controlled release of loaded agents under physiological or disease-related microenvironments.^18^ Heparin/poly-*L*-lysine nanoparticle-coated 3D-printed PLGA scaffolds facilitate bone regeneration through localized cytokine delivery and immunomodulation.^19^ GBM tumor cells and CD44-expressing fibroblasts exhibit enhanced adhesion and migration on hyaluronic acid (HA)-rich matrices through CD44/HA ligation, which temporally precedes integrin maturation and is mechanosensitive to HA hydrogel stiffness.^20^ In our previous studies, thermal nanoimprinting techniques have been used to fabricate groove structures on thermoplastic materials such as poly­(ethylene-vinyl acetate) (EVA), which can guide neuronal alignment and enhance nerve regeneration.^21^ However, EVA is hydrophobic and nonbiodegradable, limiting its biomedical applicability. To date, a hybrid technology that combines 3D printing and nanoimprinting remains an underdeveloped emerging field in biomedical applications. In recent studies, hyaluronic acid (HA)/chitosan (CHI) interfacial polyelectrolyte complex (IPC) hydrogels have been processed via a hybrid technology combining 3D printing and nanoimprinting to generate anisotropic surface topographies.^22^ Although these systems provide good structural tunability, chitosan requires prior protonation under acidic conditions, which can limit its use in direct cell-interactive biomedical applications. Therefore, there is still a need for IPC systems with improved processability under milder conditions. To address this issue, we developed an IPC system based on the electrostatic interaction between HA and poly-*L*-lysine (PLL), eliminating the need for prior protonation of PLL with acetic acid. Based on this fabrication approach, hybrid manufacturing approaches combining nanoimprinting and 3D printing, as well as gravity-compensated embedded 3D printing, were employed to accurately replicate nanoimprinted groove structures while constructing stable three-dimensional hydrogel scaffolds, demonstrating the potential of this IPC system.

Three-dimensional (3D) bioprinting has attracted significant attention for the fabrication of complex tissue constructs; however, achieving high geometric fidelity while maintaining structural stability remains challenging, especially for soft hydrogel-based bioinks.^23^ To address these limitations, support-assisted printing strategies, such as suspension-based or embedded printing,^23^ have been developed to prevent structural collapse during fabrication. In this context, gravity-compensated embedded 3D printing within a viscous suspension medium provides an effective approach by leveraging buoyancy to counteract gravitational deformation, thereby enabling the formation of freestanding and high-resolution architectures.^24^

In this study, we developed a hydrogel system based on an interfacial polyelectrolyte complex (IPC) formed between hyaluronic acid (HA) and α-poly-*L*-lysine (PLL), resulting in a highly hydrated and biodegradable matrix. In contrast to ethylene-vinyl acetate (EVA), the HA/PLL IPC hydrogel was directly utilized as a printable ink for three-dimensional (3D) fabrication. It was further integrated into a hybrid 3D printing platform that combines nanoimprinting with conventional 3D printing to generate submicrometer-scale groove architectures, thereby significantly enhancing the multiscale structural tunability of the material. Moreover, a gravity-compensated embedded 3D printing strategy was implemented, enabling deposition of the IPC hydrogel within a gel-based suspension bath without the need for additional support structures. This approach effectively minimizes gravitationally induced deformation and improves the geometrical fidelity of complex 3D constructs, underscoring the potential of such soft hydrogel systems for advanced biofabrication applications (Scheme 1).

**Scheme 1:**
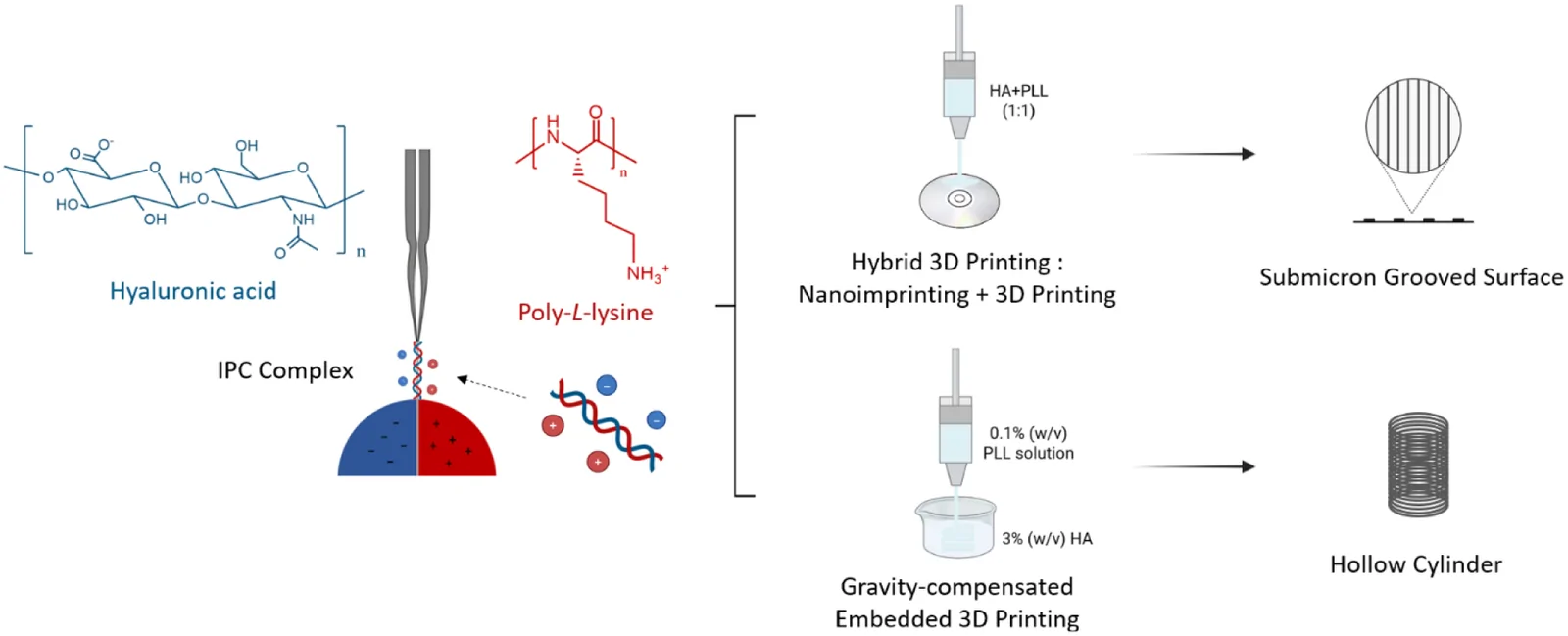
Schematic Representation of Hybrid Three-Dimensional (3D) Printing and Gravity-Compensated Embedded 3D Printing of Hyaluronic Acid/α-poly-*L*-lysine (HA/PLL) Interfacial Polyelectrolyte Complex (IPC) Hydrogels, Demonstrating an Advanced Manufacturing Strategy for Generating Anisotropic Surface Topographies with Submicrometer-Scale Grooves and Robust, Spatially Defined Three-Dimensional Fibrous Architectures

## Experimental

### Materials and Preparation

Hyaluronic acid (sodium salt from Streptococcus equi, Sigma-Aldrich; molecular weight: ∼1.5–1.8 × 10^6^ Da) was cross-linked with poly-*L*-lysine hydrochloride (Sigma-Aldrich; molecular weight: ∼15,000–30,000 Da) for hybrid 3D printing. The poly-*L*-lysine materials used in this study correspond to α-poly-*L*-lysine. Both polymers were separately dissolved in deionized water (dd-H_2_O). The negatively charged carboxylate groups (COO^–^) of hyaluronic acid interacted with the positively charged ammonium groups (NH_3_^+^) of poly-*L*-lysine through Coulombic (electrostatic) interactions, leading to the formation of an IPC complex. Additionally, a commercially available poly-*L*-lysine solution (Sigma-Aldrich; molecular weight: ∼1.5–3.0 × 10^5^ Da) was used as one of the printing materials for gravity-compensated embedded 3D printing, while a solution of hyaluronic acid (Sigma-Aldrich; molecular weight: ∼1.5–1.8 × 10^6^ Da) was used as suspension medium.

### Attenuated Total Reflection Fourier Transform Infrared (ATR-FTIR) Spectroscopy

To confirm that the membrane was formed through interactions between the NH_3_^+^ groups of PLL and COO^–^ groups of HA, FTIR (JASCO FT/IR-4700) was employed to analyze the formation of the IPC. The analysis was performed by scanning 100 times at room temperature across a range of 550 to 4000 cm^–1^.

### Electronic Circular Dichroism (ECD)

ECD analysis was employed to evaluate the absolute configuration and conformational characteristics of the samples,^25^ including the discrimination of helical structures, structural rigidity, and linkage-related information. The ECD measurements were performed using dilute polymer solutions to ensure reliable far-UV spectral acquisition. All samples were prepared to maintain absorbance values close to or below 1.0, with final HA and PLL concentrations of 0.074% (w/v) and 0.027% (w/v), respectively. IPC samples were prepared by mixing 1% (w/v) HA and 0.17% (w/v) PLL stock solutions at a 1:1 charge molar ratio. Spectra were collected using a quartz cuvette with a 1 cm optical path length across a wavelength range of 190–250 nm at a scanning rate of 100 nm/min. All experiments were performed at room temperature on a JASCO J-1700 spectropolarimeter.

### Zeta Potential

The surface charge characteristics of the particles were evaluated by measuring their zeta potential using an Anton Paar Litesizer DLS 701 based on electrophoretic mobility under an applied electric field. When an electric field is applied to the charged particles suspended in the solution, they migrate toward the electrode of opposite charge at a velocity proportional to their zeta potential. For the analysis, a fixed volume (0.5 mL) of 1% (w/v) HA was mixed with PLL stock solutions (0.17%, 0.25%, and 0.34% (w/v)) to obtain HA:PLL charge molar ratios of 1:1, 1:1.5, and 1:2, respectively.

### Dynamic Viscosity

To ensure seamless extrusion during 3D printing, shear thinning behavior is modeled to mimic how substances flow from the syringe through the needle. To evaluate whether the IPC derived from α-poly-*L*-lysine (PLL) and hyaluronic acid (HA) hydrogels possesses these shear-thinning characteristics, dynamic viscosity measurements were performed using an AMETEK Brookfield DVNext cone rheometer. The measurements were conducted at room temperature, with HA hydrogel at a concentration of 1% (w/v) and PLL hydrogel at a concentration of 0.34% (w/v).

### Hybrid 3D Printing (Nanoimprinting + 3D Printing)

Inspired by previous studies that combined 3D printing with nanoimprint lithography to fabricate micro- and nanoscale surface features using thermoplastic polycaprolactone (PCL),^26^ we extended this fabrication concept to develop a similar strategy based on IPC hydrogels for biomedical applications. In this study, IPC hydrogels prepared from HA (1% (w/v)) and PLL (0.34% (w/v)) at a charge molar ratio of 1:1 were directly printed onto a commercial DVD disc template placed inside a 3.5 cm dish at room temperature using a hybrid 3D printing approach. This setup served to maintain the geometry of the printed structure during air drying, ensuring structural stability and resulting in the formation of a uniform IPC membrane. After drying, an anisotropic surface topography with linear groove patterns was successfully replicated. Compared with thermoplastic PCL systems, IPC hydrogels offer tunable intermolecular interactions and soft-matter characteristics, providing greater flexibility and suitability for interfacial material engineering.

### Gravity-Compensated Embedded 3D Printing

A 3DDiscovery bioprinting system (regenHU) was employed to perform gravity-compensated embedded 3D printing. A pneumatic extrusion system was used to dispense a 0.1% (w/v) PLL solution through the printing nozzle into a 3% (w/v) high-concentration HA aqueous medium. By taking advantage of the rheological properties of the HA, HA matrix can act as powerful support baths or sacrificial embedding media in 3D printing by providing yield-stress environments that hold complex, overhanging, or soft constructs in place before final physical cross-linking. Under conditions in which buoyancy counterbalances gravitational forces, a near zero-gravity environment is established, enabling interfacial physical complexation to occur in three-dimensional space. This process promotes the formation of interfacial polyelectrolyte complex (IPC), which provide sufficient structural stability and shape retention for potential applications in tissue engineering. A hollow cylindrical structure was designed using BioCAD 3D modeling software. Printing parameters, including nozzle translation speed and extrusion pressure, were systematically adjusted to achieve stable fabrication of 3D architectures.

### Field-Emission Scanning Electron Microscope (FE- SEM)

FE-SEM (HITACHI SU8010) was used to observe the submicrometer groove structures of the IPC membrane. The IPC membranes were fabricated via hybrid 3D printing utilizing either commercial DVD disc or flat surfaces as nanoimprinting templates. Following lyophilization, the membranes were secured onto stubs and sputter-coated with a 40–60 nm platinum layer under a 20 mA current for 40 s prior to FE-SEM imaging.

### Measurement of the Contact Angle

A contact angle measurement system (Sindatek Model 100SB) was used to determine the contact angle (θ) to evaluate the surface hydrophilicity/hydrophobicity of the IPC membranes. Prior to measurement, all membrane samples were freeze-dried to eliminate the influence of residual moisture on the results. Deionized water (dd-H_2_O) was used as the probe liquid, and a fixed volume of 10 μL was carefully dispersed onto the membrane surface for each measurement. For each sample, six independent measurements were conducted under ambient temperature and atmospheric pressure. The average value was calculated and used for further analysis.

## Results and Discussion

### Characterization of Interfacial Polyelectrolyte Complexes (IPC)

To investigate the intermolecular interactions involved in IPC formation, ATR-FTIR measurements were performed on hyaluronic acid (HA), α-poly-*L*-lysine (PLL), and the corresponding IPC membrane. As shown in Figure 1, HA exhibits a broad band at around 3300 cm^–1^ corresponding to O–H stretching vibrations, while PLL shows an N–H stretching band at approximately 3400 cm^–1^ and a C–H stretching band at 2850 cm^–1^. In the HA spectrum, a characteristic COO^–^ asymmetric stretching band is observed at around 1600 cm^–1^, together with a C–O–C band at approximately 1030 cm^–1^. PLL exhibits amide I and amide II bands at approximately 1650 cm^–1^ and 1530 cm^–1^, respectively.

**Figure 1:**
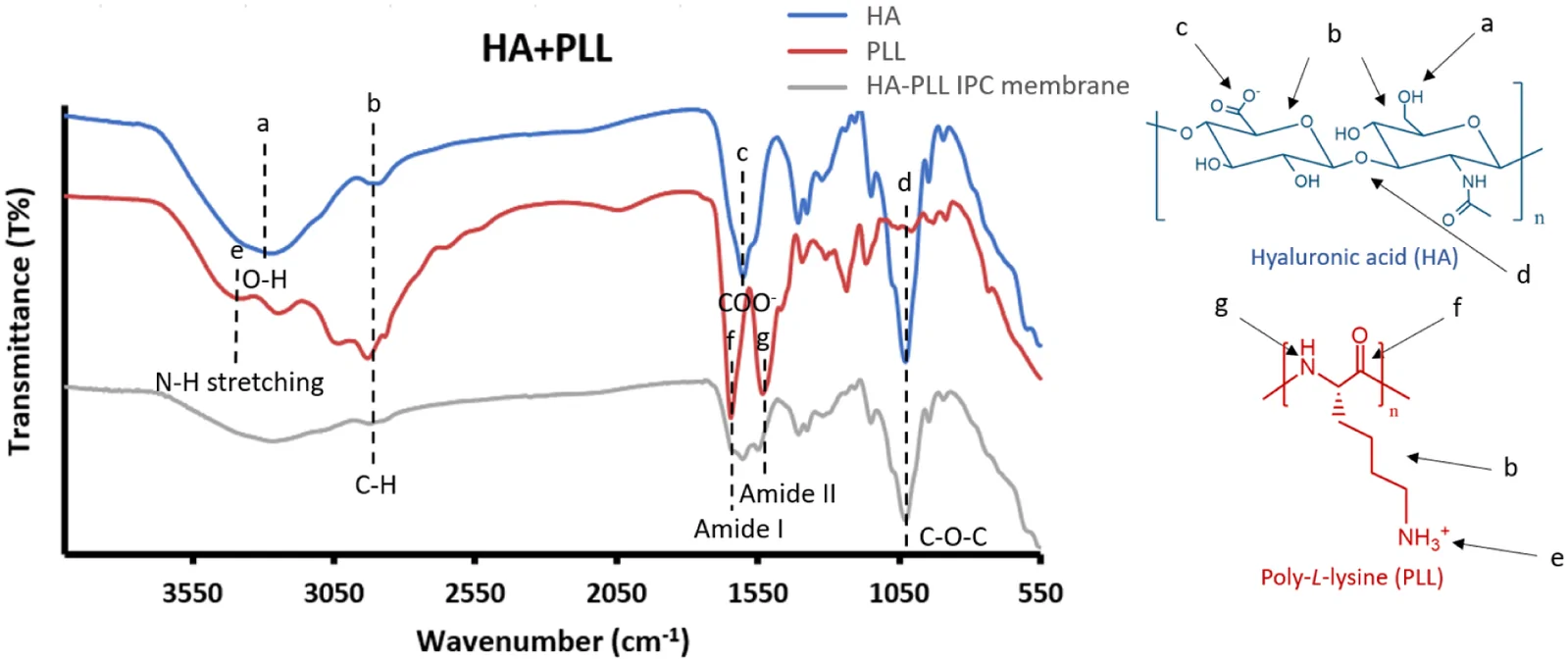
ATR-FTIR spectra of hyaluronic acid (HA, blue line), α-poly-*L*-lysine (PLL, red line), and the IPC complex used for 3D printing (1% (w/v) HA and 0.34% (w/v) PLL, mixed at an IPC charge molar ratio of 1:1) (gray line).

To evaluate changes in the chemical environments during complexation, the analysis focused on spectral profile variations rather than absolute peak intensities, as the latter may be affected by experimental variations such as differences in film thickness. Upon IPC formation, the characteristic HA COO^–^ band (1600 cm^–1^) and PLL amide I and amide II bands (1650 and 1530 cm^–1^, respectively) remained discernible but exhibited subtle alterations in their spectral profiles and band shapes compared with those of the pure components. These spectral changes suggest the formation of IPC through electrostatic interactions between the negatively charged carboxyl groups (COO^–^) of HA and the positively charged ammonium groups (NH_3_^+^) of PLL.

### Investigating the Conformational Characteristics of Interfacial Polyelectrolyte Complexes (IPC) by ECD Spectroscopy

To further investigate the conformational characteristics of the HA, PLL, and IPC hydrogel, ECD measurements were performed and converted to molar ellipticity ([θ]) (Figure 2) using the following standard equation:

1 $$\left[\theta \right]=\frac{\theta \times 100}{l\times c}$$

where θ represents the measured ellipticity (deg), l represents the optical path length (cm), and c represents the molar concentration (M) used for normalization.

**Figure 2:**
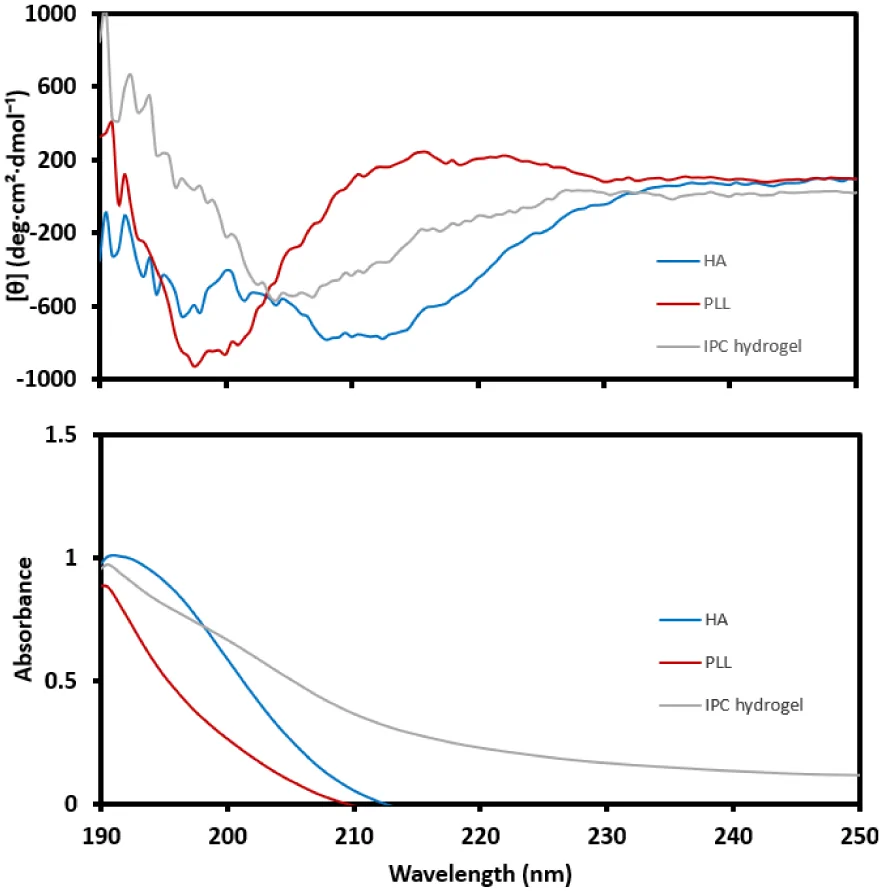
ECD and UV–vis absorption spectra of hyaluronic acid (HA), α-poly-*L*-lysine (PLL), and IPC hydrogel. The concentration of HA was 0.074% (w/v), the concentration of PLL was 0.027% (w/v), and the IPC membranes were mixed at a charge molar ratio of 1:1 with 1% (w/v) HA and 0.17% (w/v) PLL.

For HA, molar concentration was calculated from the repeating disaccharide unit, whereas PLL concentration was based on lysine residues. The IPC spectra were normalized to PLL residue concentration to evaluate backbone conformational changes upon complexation. In dilute solution, HA exhibits a negative Cotton effect near 210 nm, originating from the n-π* transition of the acetamide group’s amide chromophore, suggesting the absence of a well-defined secondary structure.^27^ In contrast, PLL exhibits a characteristic CD spectrum featuring a strong negative band near 200 nm and a weak positive band near 215 nm, arising from electronic transitions of the peptide backbone. This spectral pattern is consistent with a predominantly left-handed polyproline II (PPII)-like conformation in dilute aqueous solution.^28^ Notably, upon complexation of HA and PLL to form IPC hydrogels, the ECD spectrum shows significant deviations from those of the individual components. The characteristic signals of PLL are markedly attenuated, suggesting that changes in the magnitude and/or position of these bands indicate perturbation of the PLL chain conformation arising from electrostatic interactions between the protonated amino groups of PLL and the negatively charged carboxylate groups of HA.

### Analyzing the Charge Properties of Interfacial Polyelectrolyte Complexes (IPC) by Zeta Potential Measurements

To investigate the influence of composition on the surface charge characteristics of hyaluronic acid/α-poly-l-lysine (HA/PLL) interfacial polyelectrolyte complexes (IPC), zeta potential measurements were conducted. As shown in Figure 3, the surface charge distribution of the IPC was strongly dependent on the HA-to-PLL charge molar ratio. At a charge molar ratio of 1:1, the zeta potential distribution shifted toward negative values, indicating that under this formulation, a fraction of the anionic carboxylate (COO^–^) groups of HA remained unnaturalized by the cationic ammonium (NH_3_^+^) groups of PLL, resulting in a net negative surface charge. Upon increasing the PLL content to HA:PLL charge molar ratios of 1:1.5 and 1:2, the zeta potential shifted toward positive values, demonstrating a compositional transition from net negative to net positive surface charge with increasing PLL content. Among the tested formulations, the 1:1.5 group exhibited the highest positive zeta potential, while the 1:2 group also maintained a distinctly positive and relatively stable charge distribution. In addition, compared with the 1:1 system, the PLL-rich formulations (1:1.5 and 1:2) showed broader zeta potential distributions, which may indicate greater heterogeneity in charge compensation and local compositional organization during complex formation.

**Figure 3:**
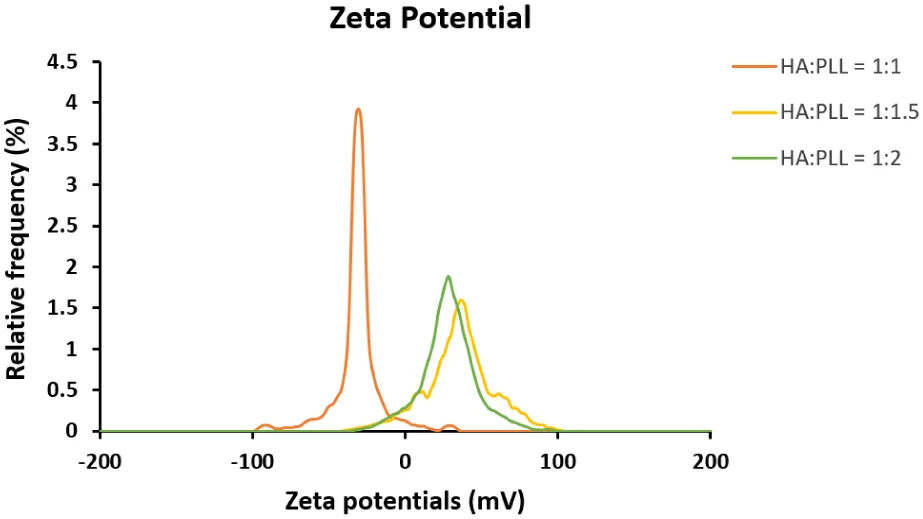
Zeta potential analysis of an IPC complex at different HA:PLL charge molar ratios. The HA concentration was fixed at 1% (w/v), while the PLL concentrations were 0.17%, 0.25%, and 0.34% (w/v), corresponding to HA:PLL charge molar ratios of 1:1, 1:1.5, and 1:2, respectively.

Collectively, these findings indicate that PLL-rich IPCs exhibit positively charged surfaces, which may facilitate electrostatic interactions with negatively charged cell membranes and, consequently, enhance their suitability for cell adhesion and related biomedical applications.^29^ In addition, the pronounced variation in zeta potential as a function of the HA-to-PLL charge molar ratio demonstrates that the surface charge of IPCs can be precisely modulated by directly adjusting the ratio of cationic to anionic components. Accordingly, the theoretical stoichiometric HA:PLL charge molar ratio (1:1) was selected as the reference formulation because it represents the theoretical stoichiometric composition for IPC formation and served as the baseline formulation throughout this study. In contrast, the PLL-rich formulations further illustrate the tunability of the IPC system and provide a basis for future optimization. This tunable surface charge characteristic offers a versatile and rational approach to accommodate diverse experimental demands and prospective biomedical applications.

### Shear Thinning of Interfacial Polyelectrolyte Complexes (IPC)

To assess the 3D printability of the IPC complex, the dynamic viscosity profiles of the hydrogels were measured. In Figure 4, the HA, PLL, and IPC hydrogels all exhibited a continuous decrease in dynamic viscosity as the shear rate increased, demonstrating characteristic shear-thinning behavior. Furthermore, the viscosity of these non-Newtonian fluids was described by fitting to the Ostwald-de Waele viscosity model using eq 2.

2 $$\eta \left(\gamma ˙\right)=m\gamma {˙}^{\left(n-1\right)}$$

where η is the viscosity, γ̇ is the shear rate, *m* is the flow consistency index, and *n* is the flow behavior index. When *n* = 1, the fluid is Newtonian; *n* < 1 and *n* > 1, represent shear—thinning and shear thickening, respectively.

**Figure 4:**
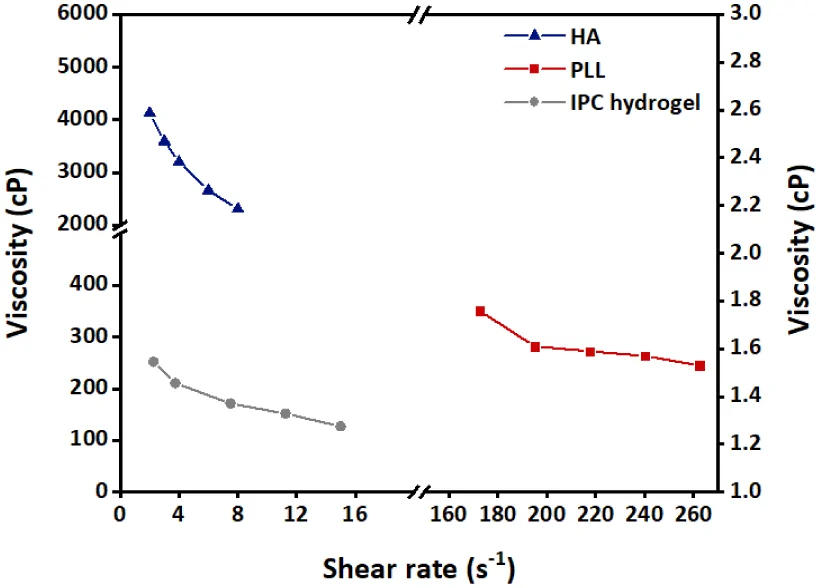
Dynamic viscosity analysis of hyaluronic acid (HA), α-poly-*L*-lysine (PLL), and IPC complexes for 3D printing. The initial concentrations of HA and PLL were 1% (w/v) and 0.34% (w/v), respectively, with the 1:1 charge molar ratio IPC hydrogel formulated using these identical components.

As shown in Table 1, the n values for the HA, PLL, and IPC hydrogels are less than 1, demonstrating a shear thinning behavior, which implies that the IPC hydrogel is suitable for 3D printing.

**Table 1:** Rheological Parameters of the Ostwald-de Waele Power Law That Characterize Flow Curves of Hyaluronic Acid (HA), α-Poly-*L*-Lysine (PLL), and IPC Hydrogel, Respectively

Sample	n	m	R^2^
**HA**	0.578	5641	0.99
**PLL**	0.646	10	0.81
**IPC hydrogel**	0.657	335	0.98

### Grooved Surface Topography via Hybrid 3D Printing of IPC Hydrogels

Taking advantage of hybrid 3D printing, the IPC complex was deposited onto a flat glass slide and a commercial DVD disc, which served as imprinting templates. After air drying, a uniform IPC membrane was obtained by peeling from the DVD template, showing iridescent diffraction colors, while the flat IPC membrane remained transparent (Figure 5a). These results demonstrate that a grooved surface topography can be successfully replicated and transferred by hybrid 3D printing of IPC hydrogels (Figure 5b). Furthermore, after initial hydration, the replicated submicron groove structures remained observable under aqueous conditions (Figure S1), qualitatively indicating that the replicated topography could be maintained after contact with water. Although both HA and PLL are inherently water-soluble materials, the physical cross-linking formed through electrostatic interactions between oppositely charged HA and PLL chains enhances intermolecular association, allowing the IPC membrane to maintain its structural integrity during exposure to water.

**Figure 5:**
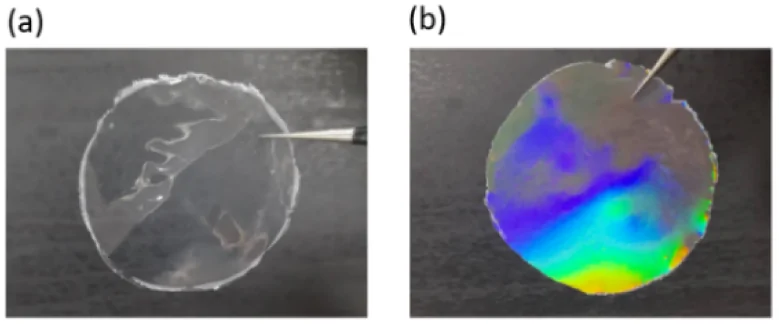
IPC hydrogel for hybrid 3D printing. Representative images of IPC membranes fabricated using (a) flat glass and (b) a commercial DVD disc as a template after air drying. (The IPC membranes were prepared by mixing 1% (w/v) HA and 0.34% (w/v) PLL at a charge molar ratio of 1:1).

### Gravity-Compensated Embedded 3D Printing of IPC Hydrogel Constructs

A cylindrical hollow model was designed using BioCAD 3D modeling software (Figure 6a) to evaluate the feasibility of gravity-compensated embedded 3D printing. The printing parameters, including a printing speed of 6 mm/s and an air pressure of 3 mPa, were optimized prior to fabrication. Using this approach, preliminary constructs were successfully fabricated from HA-PLL IPC hydrogels. The HA support matrix provided adequate support for the suspended printing process, allowing the deposited IPC filaments to maintain their position and form continuous structures. During observation, the printed IPC filaments retained their structural stability within this matrix, demonstrating the feasibility of the gravity-compensated embedded 3D printing process. As shown in Figure 6b and 6c, hollow cylindrical structures were successfully manufactured, demonstrating the potential of the technique to construct vascular-like architectures.

**Figure 6:**
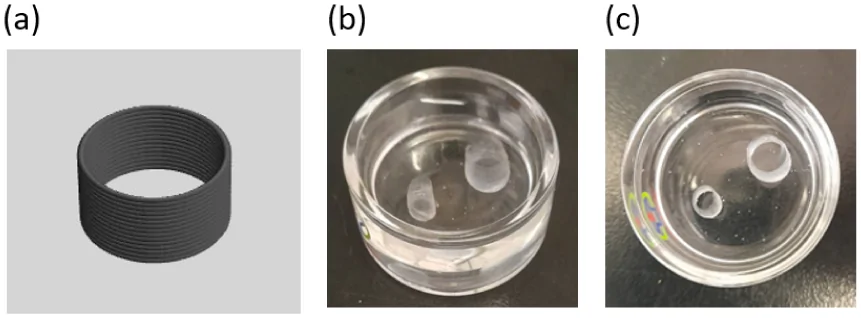
Gravity-compensated embedded 3D printing results. (a) 3D model of the printed hollow cylinder, (b) side view of the printed hollow cylinder, (c) top view of the printed hollow cylinder.

### Characterization of the Grooved Surface Topography by SEM

Scanning electron microscopy (SEM) was employed to characterize the grooved surface topography of IPC membranes replicated from commercial DVD substrates. To preserve these mechanically fragile features, the samples were subjected to lyophilization to remove residual moisture from the hydrogel matrix prior to SEM analysis. As shown in Figure 7, the pitch widths of the DVD template and the corresponding freeze-dried IPC membrane are 742 and 706 nm, respectively (Figure 7a, 7b), demonstrating that the submicrometer-scale groove structures can be effectively transferred onto the IPC membrane surface via the proposed hybrid 3D-printing-based process. Furthermore, the groove dimensions replicated on the IPC membrane exhibit high concordance with those of the original commercial DVD disc, indicating high-fidelity pattern transfer. Collectively, these findings establish a hybrid fabrication strategy that integrates soft IPC hydrogels with 3D printing and nanoimprinting techniques to generate membranes with well-defined anisotropic surface topographies. The SEM observations corroborate that the IPC membranes accurately reproduce the groove features of the templates. In contrast, no groove structures are detected on the flat (nonpatterned) membrane (Figure 7c), which serves as a control and further validates the effectiveness of the patterning procedure.

**Figure 7:**
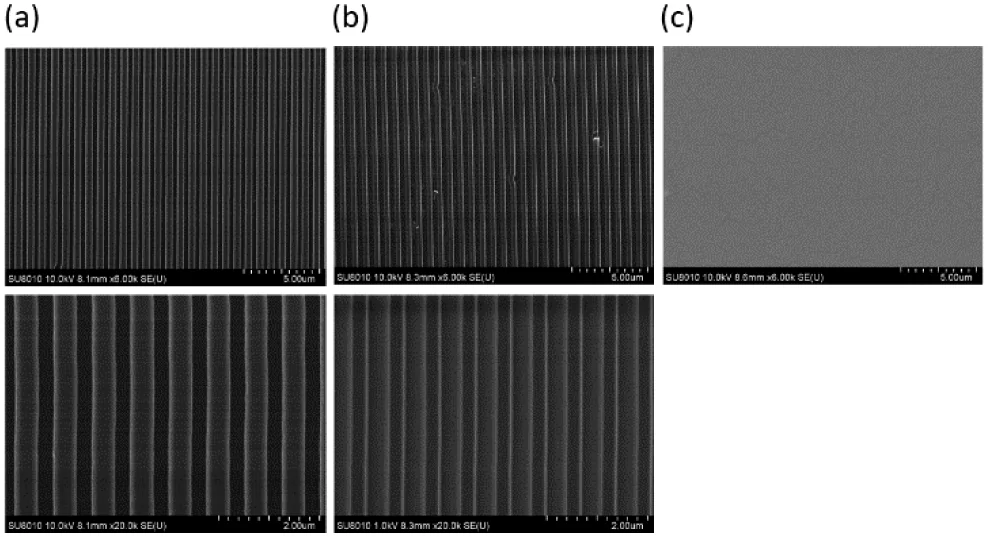
Characteristics of the surface topography of the 3D-printed IPC membranes. SEM representative images of the (a) commercial DVD disc; (b) submicron-grooved surface of an air-dried IPC membrane via DVD imprinting, and (c) flat surface of IPC membrane. (IPC membranes were replicated using female molds, followed by platinum sputtering at 20 mA for 40 s prior to SEM imaging.).

### Measurement of the Contact Angle of the Hydrophilicity for IPC Membranes

The contact angle (θ) is defined as the angle formed between tangent to a liquid droplet and the solid surface at the three-phase (solid–liquid–vapor) contact point. This parameter reflects the interfacial balance of surface tensions and is commonly used to characterize surface wettability. In general, a contact angle greater than 90° indicates a hydrophobic surface, whereas a value below 90° suggests hydrophilicity. The relationship between contact angle and interfacial tensions is described by Young’s equation (eq 3):

3 $$\gamma_{SV}=\gamma_{SL}+\gamma_{LV}\times \cos\theta$$

where $\gamma_{SV}$ represents the solid–vapor interfacial tension, $\gamma_{SL}$ represents the solid–liquid interfacial tension, and $\gamma_{LV}$ represents the liquid–vapor interfacial tension.

The test specimens comprised flat IPC membranes (Flat), DVD-patterned IPC membranes reproducing the corresponding surface microstructures (DVD), and conventional 3.5 cm culture dishes serving as the control (Control). As shown in Figure 8a, the mean static water contact angles were 76.7° for the Control, 91.3° for the flat IPC membranes, and 97.3° for the DVD-patterned IPC membranes. These measurements demonstrate the apparent hydrophobic surface character of the dried HA/PLL IPC, with the presence of surface microgrooves on the DVD-patterned substrates further enhancing this effect. This apparent surface hydrophobicity may arise from the combined contributions of (i) charge neutralization through HA-PLL ion pairing, which decreases the accessibility of strongly hydrated ionic groups; (ii) possible chain rearrangement during dehydration, resulting in preferential exposure of relatively less polar molecular segments; and (iii) the porous/rough surface morphology generated during freeze-drying, which influences the apparent wettability.

**Figure 8:**
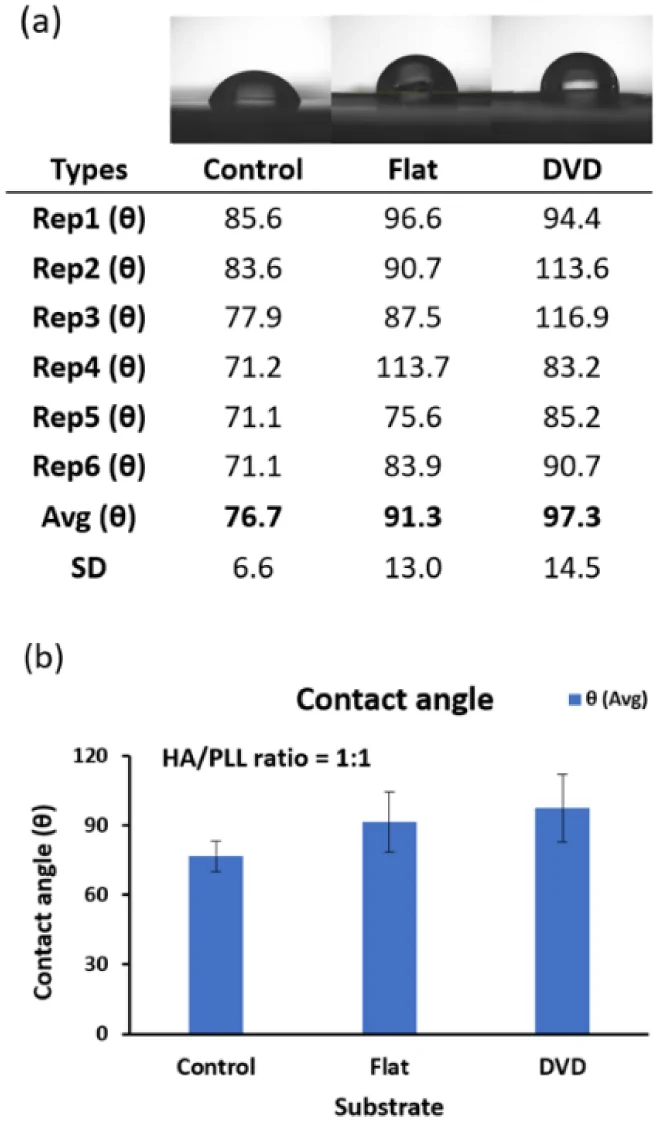
Water contact angle measurements on different substrates. Control: standard 3.5 cm culture dish; Flat: flat IPC membrane; DVD: IPC membrane with replicated DVD disc surface topography. (a) Raw data, mean values and standard deviations of water contact angles, and (b) Comparison of the bar chart of the average contact angles of water.

For subsequent cell culture studies, additional IPC membrane formulations were prepared by adjusting the HA:PLL charge molar ratios to 1:1.5 and 1:2 to increase the positive surface charge density and potentially facilitate adhesion of negatively charged cells. Contact angle measurements (Figure 9) showed hydrophobicity comparable to the original formulations, suggesting that the adjustment of HA:PLL charge molar ratios had a negligible effect on surface wettability.

**Figure 9:**
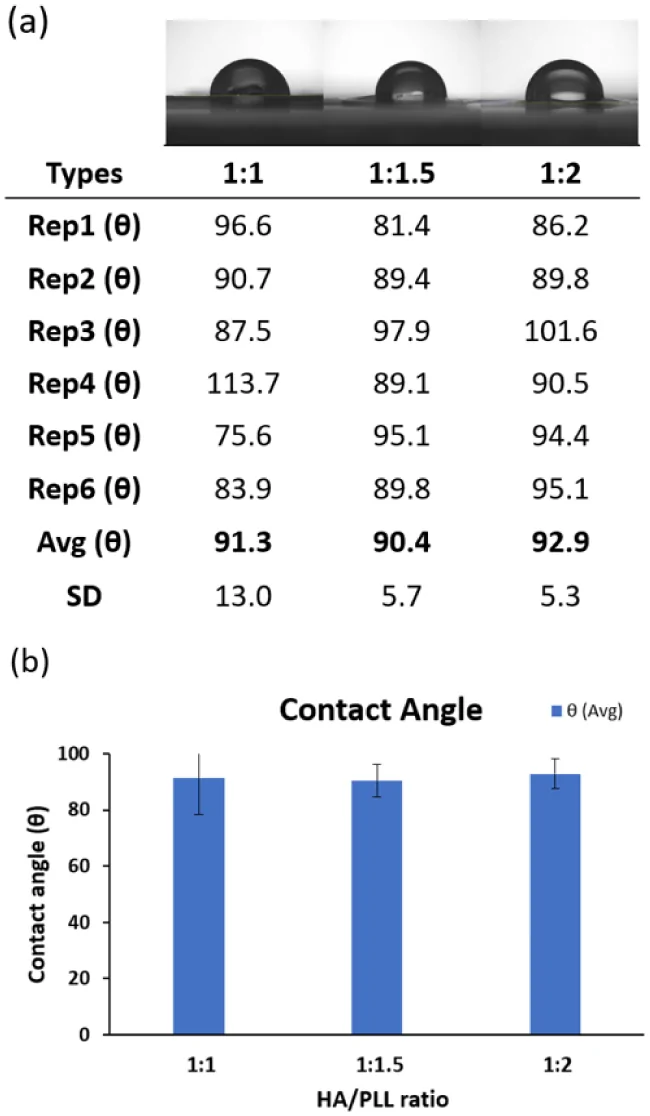
Water contact angle measurements of flat IPC membranes with different HA:PLL charge molar ratios. (a) Raw data, mean values, and standard deviations of water contact angles. (b) Bar chart comparison of average water contact angles.

## Conclusion

In this study, hybrid three-dimensional (3D) printing and gravity-compensated embedded 3D printing techniques were employed for structural fabrication. The hybrid 3D printing strategy integrates nanoimprinting with extrusion-based 3D printing: nanoimprinting was applied to generate submicrometer-scale groove features on the surfaces of soft, hydrated interfacial polyelectrolyte complex (IPC) hydrogels composed of hyaluronic acid (HA) and α-poly-*L*-lysine (PLL), whereas extrusion-based 3D printing was utilized to precisely construct three-dimensional architectures with characteristic dimensions on the order of hundreds of micrometers. In parallel, gravity-compensated embedded 3D printing enabled the direct fabrication of free-standing, mechanically stable 3D IPC hydrogel constructs without the use of sacrificial or external support materials, thereby improving overall structural fidelity and functional robustness.

Fourier-transform infrared (FTIR) spectroscopy was employed to characterize the intermolecular interactions between HA and PLL within the electrostatically assembled IPC hydrogel, and complementary structural analyses were performed to further validate the proposed interfacial complexation mechanism. The results demonstrate that this integrated fabrication strategy supports the generation of hierarchically organized hydrogels with enhanced structural stability and favorable processing characteristics. Overall, the integration of HA/PLL IPC hydrogels with hybrid and gravity-compensated embedded 3D printing represents a promising approach for the development of biomimetic scaffolds and advanced tissue-engineered constructs. SEM results demonstrated that submicrometer-scale groove patterns were successfully transferred onto hydrogel surfaces via hybrid 3D printing, and the resulting anisotropic surface topographies were verified through microscopic characterization. Moreover, three-dimensional IPC hydrogel architectures were reliably fabricated using gravity-compensated embedded 3D printing, confirming that the HA/PLL-based IPC system exhibits excellent printability and structural stability under support-free conditions. Both the grooved membranes and the 3D-printed constructs maintained their structural integrity under hydrated conditions, underscoring their suitability for the fabrication of complex biomedical architectures.

Collectively, these findings demonstrate that the combination of HA/PLL-based IPC hydrogels with hybrid 3D printing and gravity-compensated embedded 3D printing enables the production of high-precision micro/nanostructures and robust three-dimensional constructs. This integrated material–process platform offers a promising route for the development of advanced biomimetic scaffolds and tissue-engineered constructs.

## Supplementary Material



## Notes

Additional experimental details; materials and methods; supplementary characterization data; spectra; microscopy images; supporting figures and tables; Figure S1: representative optical microscopy images of IPC membranes replicated using a commercial DVD stamper under dry and hydrated conditions (PDF)
