# Supplementary material for: Hybrid 3D Printing of Interfacial Polyelectrolyte Complex Formed between Hyaluronic Acid and Poly-L-lysine Hydrogels
Source: ACS Omega. 2026 Aug 27;11(36):54512–20. doi: 10.1021/acsomega.6c06625 (PMC13584145; doi:10.1021/acsomega.6c06625)
Supplement: Supplementary file 1 [file ao6c06625_si_001.pdf]

Supporting Information

Hybrid 3D Printing of Interfacial Polyelectrolyte Complex Formed between Hyaluronic Acid and Poly-*L*-lysine Hydrogels

Yi-Chen Huang,[1] and Ming-Chia Li[1,2*]

[1] Department of Biological Science and Technology, National Yang Ming Chiao Tung University, Hsinchu 300, Taiwan

[2] Center For Intelligent Drug Systems and Smart Bio-devices (IDS2B), National Yang Ming Chiao Tung University, Hsinchu 300, Taiwan

[*]To whom correspondence should be addressed

Optical Microscopic Observation of the Grooved Surface of IPC Membranes Underwater

The surface morphology of grooved IPC membranes was examined using optical microscopy to qualitatively verify the preservation of the nanoimprinted topography upon hydration. Owing to the physical cross-linking interactions between HA and PLL, the replicated submicron groove structures remained distinguishable after immersion in dd-H_2O at room temperature (~25 °C) for approximately 5 min. However, a reduction in image contrast was observed due to hydration-induced swelling of the IPC network. (**Figure S1**).

(a) 400x_10x

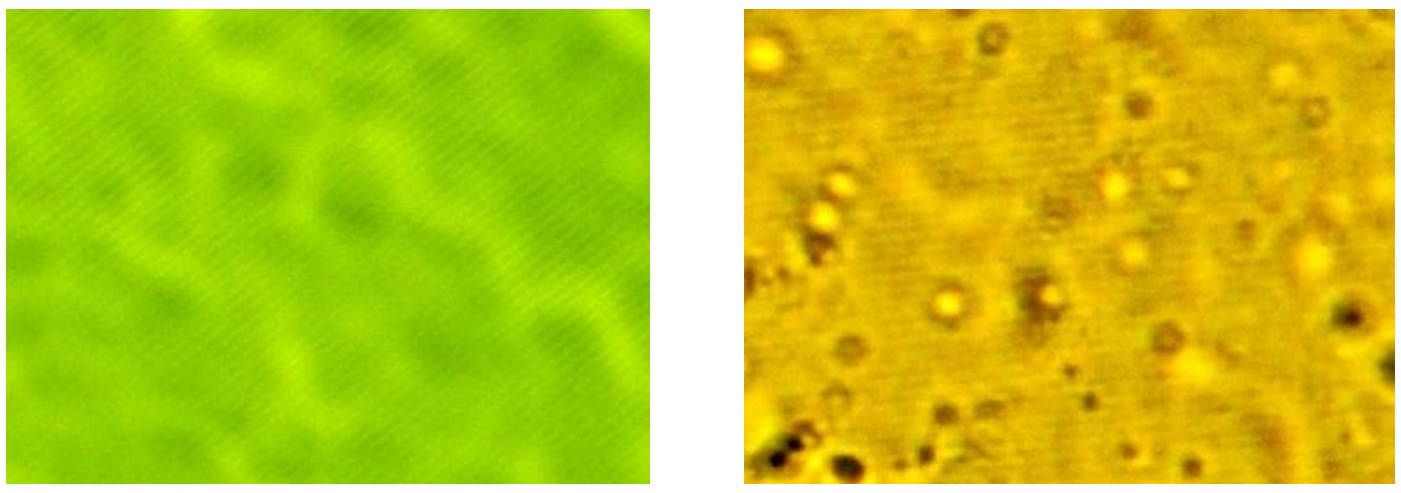

Dry state　　　　in dd-H_2O

Figure S1. Representative optical microscopy images of IPC membranes replicated using a commercial DVD disc as the plastic stamper. The groove morphology was observed at 400x magnification with an additional 10x digital zoom to compare the structural changes under dry and hydrated conditions (immersed in dd-H_2O at ~25 °C for 5 min). The IPC membranes were prepared by mixing 1% (w/v) HA and 0.34% (w/v) PLL at a charge molar ratio of 1:1. The images were processed using ImageJ for brightness and contrast adjustment to enhance the visibility of the groove structures.
